# Pharmacokinetics of isoflurane uptake and disposition in piglets

**DOI:** 10.3389/fphar.2026.1858313

**Published:** 2026-10-15

**Authors:** Che-Hao Hsu, Tso-Chou Lin, Oliver Yoa-Pu Hu, Cheng-Huei Hsiong, Shung-Tai Ho, Chih-Cherng Lu

**Affiliations:** 1 Department of Anesthesiology, Tungs’ Taichung MetroHarbor Hospital, Taichung, Taiwan; 2 Department of Anesthesiology, Tri-Service General Hospital, National Defense Medical University, Taipei, Taiwan; 3 College of Pharmacy, National Defense Medical University, Taipei, Taiwan; 4 College of Pharmacy, Taipei Medical University, Taipei, Taiwan; 5 Sinew Pharma Inc., Taipei, Taiwan; 6 Department of Anesthesiology, Kaohsiung Medical University Hospital, Kaohsiung Medical University, Kaohsiung, Taiwan

**Keywords:** disposition, inhalation anesthetics, isoflurane, pharmacokinetic, piglet, uptake

## Abstract

**Background:**

Isoflurane is a cardioprotective inhalational anesthetic widely used in cardiac surgery, but prior studies focused primarily on uptake parameters. We examined both uptake and disposition in piglets using inspiratory, end-tidal, arterial, and mixed venous concentrations.

**Methods:**

Seven piglets received a fixed inspiratory concentration of 2% isoflurane during a 2-h uptake phase, followed by a 2-h disposition phase. Inspiratory and end-tidal concentrations were measured using an infrared analyzer. Femoral arterial blood and pulmonary artery mixed venous blood were sampled to determine isoflurane concentrations via gas chromatography at 1, 3, 5, 10, 20, 30, 40, 50, 60, 80, 100, and 120 min during both uptake and disposition phases. Hemodynamic and respiratory parameters were recorded simultaneously. Body uptake and disposition rates were calculated by multiplying the difference between arterial and pulmonary artery blood concentrations by cardiac output.

**Results:**

The rates of isoflurane body uptake increased gradually during the initial 20 min, reaching 20.7 mL min^-1^, before slowly declining to 10.5 mL min^-1^ by 120 min. Continuous decreases in washout rates were observed during the subsequent disposition phase. The concentration–time curves for end-tidal, arterial blood, and pulmonary artery blood concentrations fit well with zero-order input and first-order disposition kinetics. Arterial and pulmonary artery blood concentrations were best modeled by a two-compartment system. Only 14.5% of the isoflurane accumulated during the 2-h uptake phase was eliminated during the subsequent 2-h disposition phase.

**Conclusion:**

Under a fixed inspiratory isoflurane concentration, body uptake in piglets was empirically approximated by a zero-order input process over the modeled 120-min uptake interval, whereas the subsequent 2-h disposition phase followed first-order kinetics and was best described by a two-compartment pharmacokinetic model.

## 1 Introduction

### 1.1 Isoflurane benefits in cardiac surgery

Isoflurane, an inhalational anesthetic introduced in North America in 1981, has historically been favored for cardiac anesthesia and continues to be widely utilized in current practice (O'Gara et al., 2023). Concerns regarding its potential to cause coronary steal syndrome and myocardial ischemia were effectively addressed through rigorously conducted clinical and experimental studies in the early 1990s (Agnew et al., 2002). Over time, isoflurane has been shown to exhibit preconditioning and cardioprotective effects (Tanaka et al., 2004; De Hert et al., 2005; Fu et al., 2025), contributing to improved perioperative outcomes in cardiac surgery (Bonanni et al., 2020; Abraham et al., 2023). While the pharmacokinetics of isoflurane uptake have been studied within the first 60 min following anesthesia induction in cardiac surgical patients (Lu et al., 2003a; Lu et al., 2003b), its elimination phase has been examined for only 20 min postoperatively in human studies (Lu et al., 2008; Lin et al., 2016).

### 1.2 Isoflurane characteristics and pharmacokinetic studies

Tissue/blood partition coefficients of inhalational anesthetics serve as critical indicators of wash-in and wash-out rates during induction and recovery from anesthesia, respectively. In clinical practice, isoflurane is associated with a prolonged extubation time compared to sevoflurane and desflurane (Agoliati et al., 2010). The pharmacokinetics of isoflurane uptake and elimination has been studied through respiratory and blood concentration measurements in both animal and human studies over the decades. Using inspiratory and expiratory concentrations, isoflurane demonstrates slower alveolar wash-in and wash-out, longer tissue time constants, and delayed pulmonary recovery in humans (Yasuda et al., 1991) and pigs (Yasuda et al., 1990), correlating with its higher blood/gas partition coefficient (0.94) compared to sevoflurane (0.45) and desflurane (0.35) (Yasuda et al., 1990). However, when evaluated using human blood concentrations, the blood/gas partition coefficients were determined to be 1.45 for isoflurane, 0.74 for sevoflurane, and 0.57 for desflurane (Esper et al., 2015). Furthermore, a study assessing concurrent arterial and end-tidal concentrations of isoflurane in surgical patients revealed significantly high standard deviation of variability in the concentration ratios, which underscored the clinical utility of using end-tidal concentrations to accurately predict arterial or brain concentrations of isoflurane (Frei et al., 1991). Subsequently, the following studies applied both arterial blood and respiratory concentrations to illustrate pharmacokinetics of isoflurane uptake (Lu et al., 2003a; Lu et al., 2003b; Lu et al., 2009) and elimination in humans (Lu et al., 2008; Lin et al., 2016).

### 1.3 The goals of this pharmacokinetic study

The median duration for many common surgical procedures approximates 2 hours in clinical practice (Glance et al., 2018). A study on gynecologic surgical patients reported a mean eye-opening time of 18.5 min (range 11–30 min) following the discontinuation of 2% isoflurane, administered over an average of 108 min of general anesthesia (Lin et al., 2016). Based on this, we posited that extending the elimination study period beyond 30 min would be inappropriate in surgical patients; however, a 30-min elimination period is insufficient for pharmacokinetic evaluation. Therefore, this study aimed to investigate the 120-min uptake and 120-min disposition kinetics of isoflurane in piglets, comparing inspiratory and end-tidal concentrations with femoral arterial blood and pulmonary artery mixed venous blood concentrations, providing a clearer look at total-body uptake and deposition of isoflurane to capture multi-compartmental terminal disposition kinetics. This study provides the first integrated characterization of both uptake and prolonged disposition kinetics of isoflurane in piglets under a standardized experimental setting.

## 2 Materials and methods

### 2.1 Ethics approval

This study protocol was approved by the Institutional Animal Care and Use Committee of the National Defense Medical Center in Taipei, Taiwan (NDMC-IACUC-08–028).

### 2.2 Animals

Seven domestic 5-month-old male piglets weighing 29.6 ± 7.1 kg on average were used in the study. The piglets fasted overnight with free access to water. The experiments were conducted between 8:00 a.m. and 3:00 p.m. All piglets underwent the same protocol (monitoring and anesthesia induction and maintenance).

### 2.3 Anesthetic management

All piglets were anesthetized intramuscularly with tiletamine/zolazepam (6 mg.kg^−1^; Zoletil; Virbac, France) and atropine (0.01 mg.kg^−1^; Taiwan Biotech Co., Taiwan), and maintained by continuous infusion with fentanyl (Pharmaceutical Plant of Controlled Drugs of Taiwan Food and Drug Administration, Taiwan) and propofol (Anesvan; Chi-Sheng, Taiwan) at 10–20 μg.kg^−1^.h^−1^ and 5–10 mg.kg^−1^.h^−1^, respectively, via an auricular vein. After tracheostomy in the supine position, muscle relaxation was maintained with an intravenous bolus of 0.15 mg.kg^−1^ pancuronium (Pavulon; Organon, Netherland) and followed by a continuous infusion of 0.04–0.08 mg.kg^−1^.h^−1^.

Lungs were mechanically ventilated with 40% oxygen by a conventional piston-pump ventilator (Harvard Large Animal Volume Controlled Ventilator, model 613) with a tidal volume of 8 mL∙kg^-1^ and a positive end-expiratory pressure of 5 cmH_2_O. The respiratory rate was adjusted to maintain end-tidal CO_2_ partial pressure within 35–40 mmHg.

An Opti-Q catheter (Abbott Critical Care System, Mountain View, CA) was placed into the pulmonary artery via an external jugular vein, for sampling mixed venous blood. It was connected with the Q-Vue continuous cardiac output computer (Abbott Critical Care System, Mountain View, CA) for continuous cardiac output measurements. Calibrated by the thermodilution method, iced saline was used in triplicate every 20 min. One femoral arterial catheter was inserted for blood pressure measurement and blood sampling. After 30 min of stabilization, fentanyl and propofol infusion were ceased. Forty percent oxygen gas at 5 L∙min^-1^ was passed through an anesthetic machine with an isoflurane vaporizer delivering 2% isoflurane without soda lime. The whole circuit was pre-washed for 5 min with 2% isoflurane that is equivalent to the minimum alveolar concentration (MAC) for piglets. And then the 2% isoflurane gas was connected to the Harvard animal ventilator for 2 hours. After the 2-h uptake phase, 40% oxygen gas at 5 L∙min^-1^ was connected directly to the Harvard animal ventilator for another 2 hours for the disposition phase, and anesthesia was maintained by fentanyl and propofol infusion at a dosage of 20 μg∙kg^-1^∙h^-1^ and 6–10 mg∙kg^-1^∙h^-1^ respectively. The pharmacokinetic study of isoflurane was initiated with total intravenous anesthesia using propofol and fentanyl infusions prior to the 2-h isoflurane uptake phase, and this total intravenous anesthetic regimen was restarted for the subsequent disposition phase. Hypotension, defined as 25% decrease in blood pressure from baseline, was treated with intravenous ephedrine (5 mg bolus).

The inspiratory and end-tidal isoflurane concentrations, oxygen concentration and end-tidal carbon dioxide concentration, blood pressure, heart rate and cardiac output were recorded continuously throughout the entire research period. Arterial blood and mixed venous blood gases were checked at baseline and every 60 min. In addition, the body temperature was maintained around 37 °C by heating blankets, as monitored by the Opti-Q catheter thermistor throughout the study. At the end of the study, the piglets were euthanized with an intravenous injection of pentobarbital (100 mg.kg^-1^; Easy Go; Health-Tech Pharmaceutical Co., Taiwan) and then cessation of ventilation.

### 2.4 Determination of isoflurane inspiratory and end-tidal concentrations

Anesthetic gas concentrations (%) were monitored using a multi-gas analyzer (Datex Ohmeda S/5 Compact Anaesthesia system; Datex, Helsinki, Finland). The multi-gas analyzer was calibrated prior to each experiment according to the manufacturer’s recommendations. The lower limit of detection accuracy for isoflurane was 0.2%. End-tidal values below this threshold were excluded from analysis. Isoflurane concentrations were expressed as volume percent (%) of isoflurane vapor in the carrier gas. A concentration of 1% isoflurane was defined as 1 mL of isoflurane vapor per 100 mL of gas, corresponding to 7.23 mg of isoflurane in the gas phase (Supplementary Material S1). This definition was used for subsequent pharmacokinetic analyses.

### 2.5 Blood sampling and determination of blood concentrations

Before isoflurane administration, 20 mL arterial blood was drawn through an arterial line catheter, from each piglet to determine the partition coefficients and construct calibration curves. One-ml femoral arterial and pulmonary arterial blood samples was sampled simultaneously into individual heparinized syringes at 1, 3, 5, 10, 20, 30, 40, 50, 60, 80, 100 and 120 min during each uptake and disposition period. These samples were immediately placed into a tightly sealed 10 mL glass vial. Prior to sample injection, an equal volume of gas was removed from the glass vial to minimize pressure difference. The samples were stored at 0 °C and measured within 24 h. All samples were analyzed for isoflurane concentration by gas chromatography, with a headspace sampler and a flame ionization detector according to the methods in previous studies (Supplementary Material S1) (Lu et al., 2003a; Lu et al., 2003b; Lu et al., 2008; Lin et al., 2016; Peyton et al., 2007). The experimentally determined blood/gas partition coefficient was incorporated into the preparation of calibration standards, allowing isoflurane blood concentrations to be expressed as gas-equivalent vapor concentrations for subsequent pharmacokinetic analyses.

### 2.6 Body uptake and pulmonary disposition of isoflurane

Rates of body uptake and disposition (ml of vapor∙min^-1^) through lungs are defined as follow:

$$\text{Rate of Body Uptake}=\left(\text{Aiso}-\text{PAiso}\right)\times \text{Cardiac Output}\times 10$$

$$\text{Rate of Lung-Body Disposition}=\left(\text{PAiso}-\text{Aiso}\right)\times \text{Cardiac Output}\times 10$$

Aiso and PAiso represented isoflurane concentrations in arterial and pulmonary blood. Isoflurane vapor contents in the arterial and pulmonary artery blood were expressed in ml of isoflurane vapor. The amount of volatile agent vapor deriving from 1 mL of isoflurane fluid is 195 mL (Biro, 2014). Total body uptake and disposition through lungs were estimated by the Area Under the Curve from the rate of uptake-time and rate of disposition-time curves (Lu et al., 2024).

### 2.7 Calculation of pharmacokinetic parameters

One-, two-, and three-compartment models with zero order input and first order elimination kinetics were fitted to femoral arterial and pulmonary artery-time curves by using WinNonlin 5.2 (Pharsight Corporation, Mountain View, CA, USA). A more explicit description of the fitted model additional details of the model structures implemented in WinNonlin have also been expressed in the Supplementary Material (S2 Compartment models). The selection of the best-fit model was made based on the Akaike information criterion (Yamaoka et al., 1978). The model that gives the lowest Akaike information criterion value is considered the best fit (Takeda et al., 2000). Model selection was primarily guided by two considerations. First, the Akaike Information Criterion (AIC) was used as the principal metric, with lower AIC values indicating a better balance between model fit and complexity. Second, although increasing the number of fitting parameters typically leads to a reduction in AIC, it also raises the risk of overfitting. Therefore, when the inclusion of additional parameters did not result in a significant decrease in AIC, the model with fewer parameters was preferred in accordance with the principle of parsimony. Total body uptake doses were used to calculate pharmacokinetic parameters. Isoflurane concentrations and the derived pharmacokinetic parameters were reported as mean (±SD).

### 2.8 Statistical analysis

The one-way ANOVA with Scheffé's *post hoc* test was applied only to derived pharmacokinetic parameters, whereas serial physiological variables are presented descriptively without formal longitudinal statistical comparisons.

## 3 Results

The blood–gas partition coefficient of isoflurane before body uptake phase was 0.97 ± 0.01. As presented in Table 1, the average ratios of arterial over end-tidal concentrations ranged between 0.85 and 0.88 after 5 min during the uptake phase, while the average ratios of arterial blood to pulmonary artery blood concentrations decreased gradually from 0.87 to 0.70 during the disposition phase. The isoflurane uptake rates were near constant in the initial 30 min, with a peak concentration 20.7 ± 9.3 mL.min^-1^ at 20 min. The disposition rates decreased gradually from 6.3 ± 3.7 mL.min^-1^ at 1 min to 2.8 ± 1.5 mL.min^-1^ at 5 min. The total body uptake (determined from the difference in concentrations between the arterial and mixed venous blood) over 2 h was 7.5 ± 2.0 mL isoflurane fluid by blood and tissue. The total amount of isoflurane eliminated over 2 h, calculated from the arteriovenous concentration difference, was 1.1 ± 0.4 mL, representing 14.5% ± 4.5% of total uptake.

**TABLE 1:** Inspiratory, end-tidal, arterial blood, and pulmonary artery blood concentrations of isoflurane and rates of body uptake and disposition phases.

Concentration/Cardiac output	Uptake phase (min)								Disposition phase (min)							
​	1	3	5	10	20	30	60	120	1	3	5	10	20	30	60	120
Inspiratory, %	1.82 (0.12)	1.92 (0.07)	1.99 (0.07)	2.02 (0.04)	2.02 (0.04)	2.03 (0.05)	2.03 (0.05)	2.04 (0.05)	0	0	0	0	0	0	0	0
End-tidal (ET), %	0.72 (0.22)	1.08 (0.19)	1.19 (0.18)	1.47 (0.11)	1.56 (0.11)	1.58 (0.10)	1.61 (0.10)	1.75 (0.05)	0.72 (0.14)	0.51 (0.05)	0.44 (0.05)	0.34 (0.05)	0.24 (0.03)	0.18 (0.03)	--	--
Arterial blood (A), %	0.68 (0.18)	0.90 (0.16)	1.04 (0.16)	1.26 (0.08)	1.32 (0.10)	1.37 (0.09)	1.37 (0.14)	1.54 (0.11)	0.73 (0.08)	0.58 (0.05)	0.49 (0.03)	0.39 (0.04)	0.28 (0.04)	0.22 (0.04)	0.14 (0.03)	0.08 (0.03)
Pulmonary artery blood (PA), %	0.25 (0.11)	0.52 (0.15)	0.65 (0.14)	0.84 (0.16)	0.96 (0.16)	1.04 (0.09)	1.14 (0.10)	1.27 (0.14)	0.82 (0.10)	0.67 (0.06)	0.56 (0.06)	0.46 (0.05)	0.33 (0.04)	0.28 (0.04)	0.19 (0.04)	0.12 (0.04)
A/ET during uptake	0.99 (0.27)	0.83 (0.06)	0.88 (0.11)	0.86 (0.04)	0.85 (0.07)	0.87 (0.04)	0.85 (0.08)	0.88 (0.06)	--	--	--	--	--	​	--	--
PA/A during uptake	0.37 (0.16)	0.57 (0.11)	0.63 (0.07)	0.67 (0.11)	0.73 (0.07)	0.76 (0.04)	0.83 (0.05)	0.82 (0.06)	--	--	--	--	--	​	--	--
ET/A during disposition	--	--	--	--	​	--	--	--	0.98 (0.10)	0.88 (0.02)	0.89 (0.06)	0.87 (0.06)	0.86 (0.06)	0.82 (0.06)	--	--
A/PA during disposition	--	--	--	--	​	--	--	--	0.91 (0.15)	0.88 (0.14)	0.87 (0.09)	0.86 (0.06)	0.85 (0.05)	0.80 (0.05)	0.74 (0.10)	0.70 (0.11)
Cardiac output, l.min^-1^	3.8 (0.7)	4.0 (0.7)	4.2 (0.9)	4.5 (1.6)	5.5 (2.5)	5.3 (2.5)	4.4 (1.1)	3.5 (0.4)	3.7 (0.5)	3.7 (0.4)	4.1 (0.6)	3.8 (0.4)	4.2 (0.5)	4.3 (0.7)	3.6 (0.9)	3.5 (0.7)
*Rates of body uptake/disposition in vapor, ml.min^-1^	15.2 (7.3)	15.1 (3.9)	15.5 (4.0)	18.8 (4.7)	20.7 (9.3)	17.9 (8.2)	10.4 (4.5)	10.5 (2.7)	6.3 (3.7)	4.8 (1.5)	2.8 (1.5)	3.0 (0.7)	2.5 (0.9)	2.4 (0.8)	1.8 (0.8)	1.1 (0.5)

Data are presented as means (standard deviations).

A, arterial blood concentration, % in vapor; ET, end-tidal concentration, % in vapor; PA, pulmonary artery blood concentration, % in vapor.

* Rate of body uptake in vapor [ml.min-1] = 10 x (A− PA) [ml.dl-1] × cardiac output [l.min-1].

Rate of body disposition in vapor [ml.min^-1^] = 10 × (PA − A) [ml.dl^-1^] × cardiac output [l.min^-1^].

The estimated total uptake and disposition of isoflurane fluid in blood was 7.5 ± 2.0 mL and 1.1 ± 0.4 mL, respectively, by Henry’s law. (Biro, 2014).

The amount of volatile agent vapor deriving from 1 mL of isoflurane fluid is 195 mL. (Biro, 2014).

Figure 1 depicts the concentration–time curves and an exponential decline with a constant first-order disposition rate on a semilogarithmic plot in 2 h. Figure 2 illustrates the body uptake and disposition rates of isoflurane during the 2-h uptake phase and the subsequent 2-h disposition phase.

**FIGURE 1:**
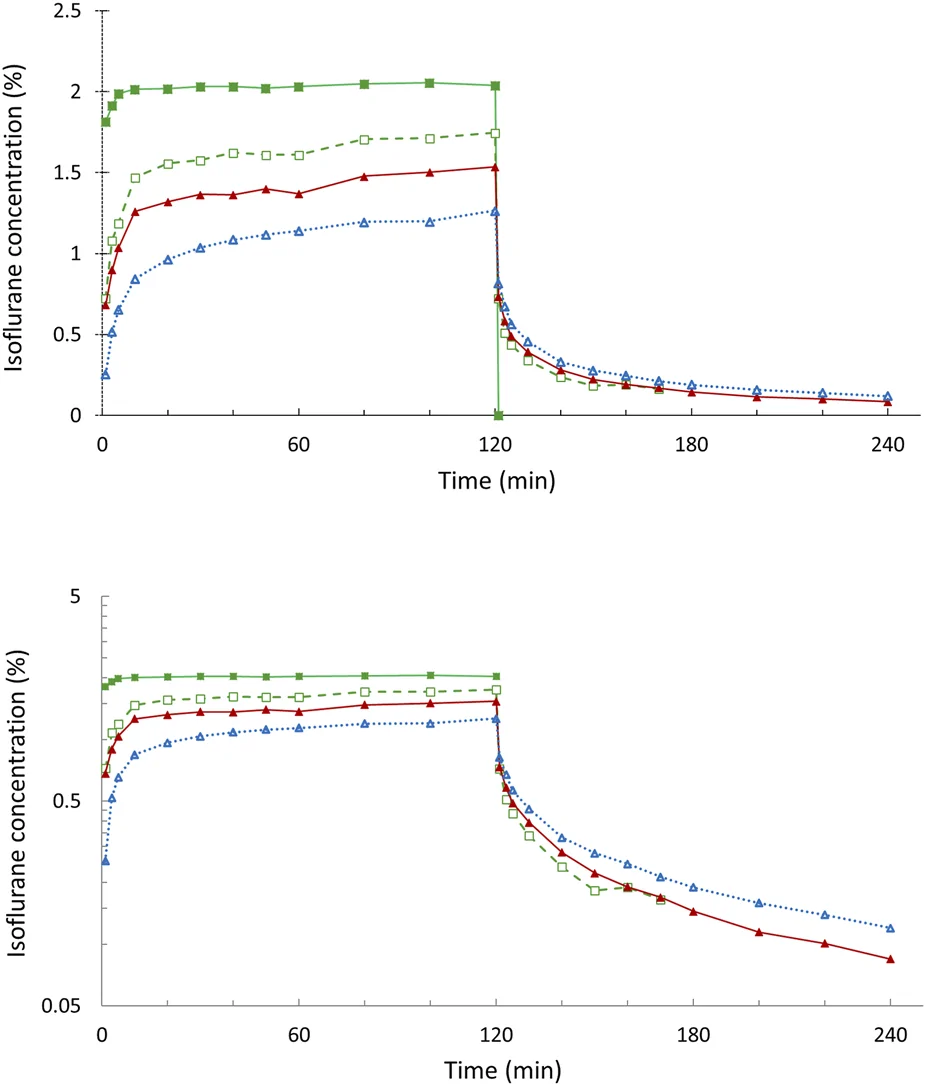
Mean isoflurane concentration–time curves during 2-h uptake and 2-h disposition (upper panel). The semi-logarithmic plot (lower panel) illustrates that the measured uptake phase was empirically approximated by a zero-order input process over the modeled 120-min interval, whereas the disposition phase followed first-order kinetics. Inspiratory (filled squares), end-tidal (open squares), arterial (filled triangles), and mixed venous (open triangles) isoflurane concentrations are shown.

**FIGURE 2:**
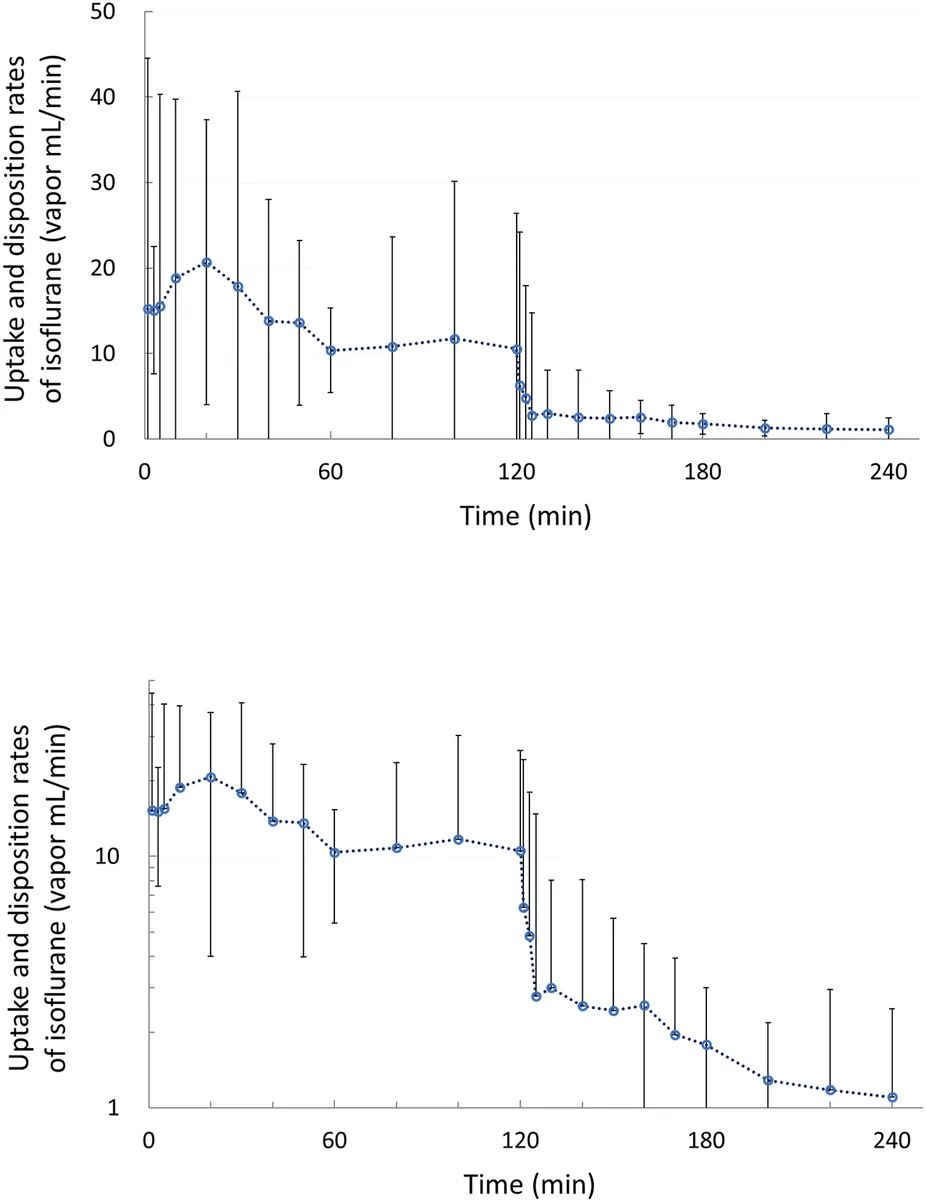
Body uptake and disposition rates of isoflurane during 2-h uptake and 2-h disposition (upper panel). The semi-logarithmic plot (lower panel) demonstrates that the measured uptake phase was empirically approximated by a zero-order input process over the modeled 120-min interval, whereas the disposition phase followed first-order kinetics.

The hemodynamic and respiratory variables, including heart rate, mean arterial pressure, end-tidal carbon dioxide levels, and minute ventilation, are summarized in Table 2. No vasopressors, including ephedrine, were required or administered to any animal during the experimental procedures. No significant differences in hemodynamic or respiratory variables were observed throughout the uptake and disposition phases.

**TABLE 2:** Hemodynamic and respiratory variables.

​	Uptake phase (min)								Disposition phase (min)							
Variables	1	3	5	10	20	30	60	120	1	3	5	10	20	30	60	120
Heart rate, beats per min	109 (30)	110 (48)	110 (48)	118 (49)	117 (45)	125 (43)	118 (64)	115 (24)	111 (21)	109 (20)	113 (17)	121 (22)	117 (23)	105 (18)	85 (27)	83 (38)
Mean arterial pressure, mmHg	104 (38)	110 (38)	96 (41)	91 (33)	72 (12)	70 (22)	74 (26)	64 (6)	81 (16)	83 (9)	93 (16)	104 (7)	108 (15)	101 (20)	85 (17)	82 (24)
End-tidal CO_2_, mmHg	40 (4)	39 (5)	38 (6)	37 (4)	37 (4)	38 (4)	41 (3)	37 (4)	36 (3)	36 (3)	37 (6)	39 (3)	39 (2)	39 (3)	36 (4)	36 (3)
Tidal volume, ml	291 (76)	306 (88)	320 (100)	313 (82)	313 (73)	301 (78)	301 (76)	320 (90)	324 (77)	331 (93)	327 (97)	319 (69)	317 (77)	317 (82)	323 (80)	321 (67)
Respiratory rate, per min	10.7 (1.9)	10.4 (1.1)	10.4 (0.4)	10.0 (0)	10.0 (0)	10.0 (0)	10.0 (0)	10.0 (0)	10.0 (0)	10.0 (0)	10.0 (0)	10.0 (0)	10.0 (0)	10.0 (0)	10.0 (0)	10.0 (0)
Minute ventilation, l.min^-1^	3.1 (1.0)	3.2 (1.1)	3.4 (1.3)	3.1 (0.8)	3.1 (0.7)	3.0 (0.8)	3.0 (0.8)	3.2 (0.9)	3.2 (0.8)	3.3 (0.9)	3.3 (1.0)	3.2 (0.7)	3.2 (0.8)	3.2 (0.8)	3.2 (0.8)	3.2 (0.7)

The data are presented as means (standard deviations).

Table 3 demonstrates the fitting parameters of the end-tidal, arterial blood, and pulmonary artery blood concentrations during the uptake and disposition phase. The data fitted well to a biexponential curve for arterial blood and pulmonary artery blood concentration–time curves.

**TABLE 3:** The fitting parameters of the end-tidal, arterial blood, and pulmonary artery blood concentrations during the uptake and disposition phase.

Parameters	End-tidal concentration	Arterial blood concentration	Pulmonary artery blood concentration
A_1_ (mg.dl^-1^)	−7.815 (1.143)^a^	−7.114 (0.954)^b^	−5.302 (1.436)
B_1_ (mg.dl^-1^)	−4.388 (0.913)	−3.873 (0.701)	−4.093 (0.908)
Alpha (1.min^-1^)	1.39 (0.29)^a^	1.24 (0.42)^b^	0.48 (0.16)
Beta (1.min^-1^)	0.057 (0.020)^b^	0.026 (0.008)	0.020 (0.010)
Alpha half-life (min)	0.5 (0.1)^a^	0.6 (0.2)^a^	1.6 (0.5)
Beta half-life (min)	14.2 (6.5)^a^	29.2 (8.4)^b^	45.3 (14.1)
Mean residence time (min)	7.4 (2.2)^a^	15.1 (3.8)^a^	28.6 (7.0)
K_21_ (1.min^-1^)	0.0904 (0.0419)^b^	0.0405 (0.0177)	0.0340 (0.0263)
V (dl)	0.0056 (0.0022)^a^	0.0074 (0.0035)^a^	0.0239 (0.0061)
Maximal concentration (mg.dl^-1^)	12.17 (0.55)^b^	10.75 (0.45)	8.75 (0.59)
Area under the curve (mg.dl^-1^ × min)	1464 (64)^b^	1319 (61)^c^	1125 (100)

Values are presented as means (± standard deviations).

Biexponential equation: Y(T) = A1*(EXP (-ALPHA*T)-EXP (-ALPHA*TSTAR)).

+B1*(EXP (-BETA*T)-EXP (-BETA*TSTAR)), where Y represents the end-tidal, arterial blood, and pulmonary artery blood concentrations; TI, infusion length, TSTAR = T–TI, for T > TI (Disposition phase); TSTAR, 0 for T ≤ TI (Uptake phase).

$A_{1}=\frac{D}{TI\left(V\right)}\times \frac{\left(K_{21}-Alpha\right)}{\left(Alpha-Beta\right)Alpha}$

$B_{1}=\frac{-D}{TI\left(V\right)}\times \frac{\left(K_{21}-Beta\right)}{\left(Alpha-Beta\right)Beta}$

a *P* < 0.001; b *P* < 0.01; c *P* < 0.05, compared with the significance levels of the pulmonary artery blood concentrations.

Figure 3 shows that the end-tidal, arterial blood, and pulmonary artery blood concentration–time profiles were adequately fitted by an empirical pharmacokinetic model in which the uptake phase was approximated by a zero-order input process during the modeled 120-min interval, whereas the disposition phase followed first-order kinetics. The observed concentrations closely matched the corresponding model-predicted concentrations. The model-fitting parameters and concentration–time profiles are also presented in clinically reported units (%) in Supplementary Material S4.

**FIGURE 3:**
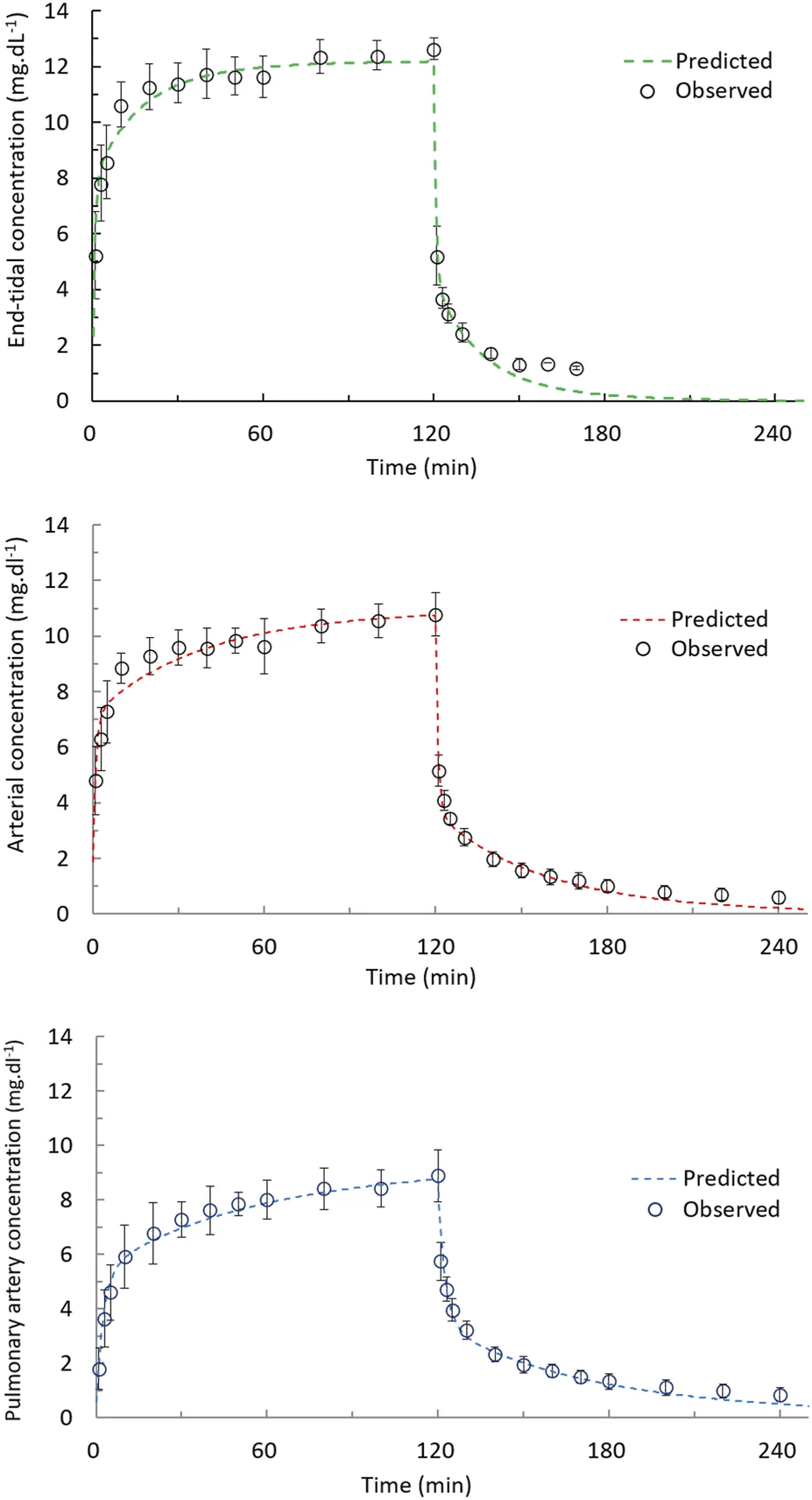
Comparison of observed (open circles) and model-predicted (dotted lines) isoflurane concentration–time curves obtained using an empirical pharmacokinetic model. The uptake phase was approximated by a zero-order input process over the modeled 120-min interval, whereas the disposition phase followed first-order kinetics.

## 4 Discussion

### 4.1 The major findings

This pharmacokinetic study investigated the uptake and disposition of isoflurane during 2-h uptake and 2-h disposition phases in piglets. With concomitant measurement of inspiratory, end-tidal, arterial, and mixed venous blood concentrations, body uptake rates approached a peak at 20 min and subsequently declined gradually. Body uptake was empirically approximated by a zero-order input process during the modeled 120-min uptake phase, whereas the disposition phase followed first-order kinetics and was adequately described by a two-compartment pharmacokinetic model. Only 14.5% of uptake isoflurane was eliminated through lungs at the end of 2-h disposition.

### 4.2 Cardioprotection with volatile anesthetics against ischemia-reperfusion injury

Experimental evidence indicates that clinical concentrations of volatile general anesthetics protect the myocardium from ischemia and reperfusion injury (De Hert et al., 2005). Volatile usage during cardiac surgery is beneficial for attenuation of myocardial damage, along with the reduced intensive care unit and hospital length of stay (Abraham et al., 2023). In a survey of cardiac anesthesiologists published in 2023, a majority of respondents indicated that isoflurane was the most frequently selected primary agent for maintenance of anesthesia for the prebypass, bypass, and postbypass periods (57%, 62%, and 50%, respectively) (O'Gara et al., 2023). Ease of use was the most frequently selected reason for administering isoflurane and sevoflurane for each period. Most respondents (74%) think that isoflurane has organ-protective properties, and 72% believed that anesthetic choice contributes to patient outcome (O'Gara et al., 2023). The previous animal studies published in the mid-1980s suggested that isoflurane may be capable of producing an abnormal redistribution of coronary blood flow away from ischemic toward normal myocardium, a so-called “coronary steal” phenomenon (Agnew et al., 2002). Subsequent investigations conducted in animal models and humans with coronary artery disease did not reduce or abnormally redistribute coronary collateral blood flow, shown to be relatively weak coronary vasodilators that are incapable of causing coronary steal under the vast majority of clinical conditions (Tanaka et al., 2004). In this study, we conducted a pharmacokinetic investigation of isoflurane in an animal model to determine its pharmacokinetic parameters, which will facilitate future pharmacodynamic investigations into its cardioprotective effects.

Furthermore, the molecular mechanisms underlying anesthetic preconditioning and postconditioning have been investigated to translate these protective strategies into volatile anesthetic-mediated cardioprotection in patients undergoing cardiac surgery (Fu et al., 2025), thereby contributing to a preference for isoflurane in clinical cardiac anesthesia (O'Gara et al., 2023).

### 4.3 Two-compartment model of disposition

The process of emergence from general anesthesia with inhalational anesthetics is primarily driven by pulmonary exhalation and anesthetic disposition (Vincent et al., 2025). Isoflurane is subject to little or no biotransformation (<0.2%) and percutaneous losses (0.35% of uptake) to affect kinetic studies (Holaday et al., 1975). Five-compartment models was reported to fit the washout curves up to 5–9 days in healthy patients (Carpenter et al., 1986). However, the median duration for the majority of common surgical procedures is approximately 2 hours and the stay in the post-anesthesia care unit is less than 2 hours in clinical practice (Glance et al., 2018). Therefore, it is reasonable that two distinct disposition components of inhalation anesthetics were identified in a clinical setting (Wissing et al., 2000), with an initial rapid 5-min washout of functional residual capacity followed by a slow 15-min constant diffusion of isoflurane from arterial blood to alveolar space (Lu et al., 2008). Accordingly, based on the model selection criteria and the evaluation of the residual plots (Supplementary Material S3), the three-compartment model did not provide a statistically or practically significant improvement over the two-compartment model. Therefore, the two-compartment model was selected as the final model for fitting the arterial blood isoflurane concentration (Aiso), pulmonary arterial blood isoflurane concentration (PAiso), and end-tidal isoflurane concentration (CEiso), as it achieved an appropriate balance between goodness-of-fit and model parsimony. In the present piglet study, the agreement between predicted and experimental data (Figure 3) supports the two-compartment model of isoflurane disposition within 2 hours.

### 4.4 Uptake and disposition of inhalation anesthetics

The kinetics of wash-in and wash-out of inhalational anesthetics are primary determinants of the speed of anesthetic induction and emergence. Under conditions of a constant inspired concentration and stable cardiac output, isoflurane uptake approaches a steady state after an initial 4–5-min wash-in period (Hendrickx et al., 1997; Lu et al., 2003b). The rate of body uptake depends on the blood/gas partition coefficient of inhalational anesthetics across the alveolar membrane. Isoflurane has a higher blood/gas partition coefficient than sevoflurane and desflurane in both pigs (0.94 vs. 0.45 and 0.35, respectively) and humans (1.45 vs. 0.74 and 0.57, respectively) (Yasuda et al., 1990; Esper et al., 2015). The concentration curves have been approximated to be a zero-order input approximation during the modeled uptake phase in the current study. Immediately after the cessation of administration, alveolar isoflurane is washed out of lungs within the initial 5-min wash-out period. The following elimination of inhalation anesthetics from pulmonary artery blood to alveoli correlates with the blood/gas partition coefficient and the tissue/blood partition coefficient from tissues to pulmonary artery blood, such as fat/blood partition coefficient of isoflurane 47.5, sevoflurane 44.9, and desflurane 27.2 in humans (Yasuda et al., 1989). With relative slow elimination of isoflurane, the time taken for eliminating 99.9% of isoflurane from the brain was up to 71 h (Lockwood, 2010). In the current piglet study, only an estimated 14.5% of the absorbed isoflurane was eliminated after 2 h of disposition. Therefore, substantial amounts of isoflurane likely remain in the body for hours represent the pharmacokinetic characteristics of isoflurane disposition following 120 min of isoflurane cessation (Lin et al., 2016; Agoliati et al., 2010; Frink et al., 1992).

### 4.5 Clinical relevance of isoflurane pharmacokinetic study in piglets

The present study provides an integrated characterization of the uptake and prolonged disposition kinetics of isoflurane in piglets using simultaneous measurements of inspiratory, end-tidal, arterial, and mixed venous concentrations. The findings demonstrate that body uptake can be empirically approximated by a zero-order input process during the modeled uptake phase, whereas the subsequent disposition phase follows first-order kinetics and is adequately described by a two-compartment pharmacokinetic model. Furthermore, the observation that only 14.5% of the absorbed isoflurane was eliminated during the first 2 hours after discontinuation quantitatively demonstrates the prolonged tissue retention of isoflurane.

### 4.6 Limitations

First, the lower limit of detection accuracy for isoflurane end-tidal concentration was 0.2%, which may limit measurement precision at low concentrations after 20 min of the disposition phase. Therefore, more sensitive methods, such as gas chromatography, are warranted to better define the relationship between end-tidal and arterial concentrations. Second, isoflurane exhibits high tissue solubility, particularly reflected by its fat/blood partition coefficient, and may undergo multi-phase disposition. Accordingly, the 2-h uptake and 2-h disposition periods used in this study may be insufficient to fully characterize disposition kinetics. Third, the anesthetic depth, ventilation, hemodynamics, and infusion protocols were maintained as consistently as possible throughout the uptake and disposition phase to minimize physiological variability.

## 5 Conclusion

With a fixed inspired concentration, body uptake was empirically approximated by a zero-order input process during the modeled 120-min uptake phase, whereas the subsequent disposition phase followed first-order kinetics. The concentration-time curves of arterial blood and mixed venous blood were parallel and the pharmacokinetic properties were compatible. At the end of the 2-h disposition phase, limited isoflurane is still retained in the body. These findings extend previous human isoflurane pharmacokinetic studies and our earlier piglet desflurane model, providing fundamental pharmacokinetic parameters that have practical implications for optimizing isoflurane delivery during the induction, maintenance, and emergence periods for veterinary surgical practice and future translational studies.

## Data Availability

The raw data supporting the conclusions of this article will be made available by the authors, without undue reservation.
