# Supplementary material for: Pharmacokinetics of isoflurane uptake and disposition in piglets
Source: Front Pharmacol. 2026 Oct 15;17:1858313. doi: 10.3389/fphar.2026.1858313 (PMC13716762; doi:10.3389/fphar.2026.1858313)
Supplement: Supplementary file 1 [file DataSheet1.pdf]

Supplementary Materials

S1 Appendix

I. Gas chromatography (GC) conditions for isoflurane quantification

The HP 6890 series GC system (Hewlett-Packard, Wilmington, DE, USA) consisted of a Headspace Sampler (Agilent G1888), an oven, a flame ionization detector, and an integrator. The oven temperature was raised from 40°C to 200°C at a rate of 15°C min^{-1}, held for 4.33 minutes, over a 15-minute cycle. Both injection and detection temperatures were set at 250ºC with the inlet pressure of 349 kPa. The injection was performed in the direct injection mode. The carrier gas (helium) flow was 25.0 ml.min^{-1}, and separation was achieved using a capillary column (HP-5; 30.0 m × 0.32 mm internal diameter, 0.25 µm film thickness; Restek, Bellefonte, PA, USA). An integrator and a datum acquisition system were provided by Agilent CHEMOSTATION software.

II. Primary calibration curve with known concentrations of isoflurane gas:

The calibration curve was obtained by injecting different concentrations of isoflurane into the gas chromatogram using a gas-tight Hamilton syringe. To obtain standard gas concentrations, five 550 ml glass bottles, each with a turnable stopcock and Teflon septum, were used. After flushing each bottle with nitrogen for 6 minutes, the septum was pierced with a syringe needle and 2, 5, 10, 20, or 50 µl of liquid isoflurane were injected into the glass bottles at 4°C. The calculated volume percent of isoflurane was plotted against the measured GC counts.

III. Determination of isoflurane blood / gas ($\lambda_{b/g}$) partition coefficients for each piglet:

The blood⁄gas partition coefficients for isoflurane can be calculated if concentrations of isoflurane in the equilibrated sample headspace were determined by using the primary calibration curve. The blood⁄gas partition coefficients of isoflurane were determined by the two-stage method *[Smith et al, 1997]* as follows:

A 2-ml blood sample with unknown isoflurane concentration was added into the first 10-ml vial (with 2 ml of gas removed before injection), sealed with a Teflon septum and then incubated at 37°C for 30 minutes. The isoflurane concentration in the headspace of the first vial (C1) was determined by gas chromatography. One ml of sample was withdrawn immediately from the first vial and

transferred to the second 10-ml vial (with 1 ml of gas removed before injection) sealed with a Teflon septum and then incubated at 37°C for another 30 minutes. The isoflurane concentration in the headspace of the second vial (C2) was again determined by gas chromatography. Thus, the λ can be calculated by the following equation:

$$\lambda = \frac{9 \times C2}{C1 - C2}$$

IV. Calculation of isoflurane contents in gas phase, in blood phase by known isoflurane blood gas partition coefficients (λ):

According to the following isoflurane physical properties:

MW = molecular weight of isoflurane (184.5 g);

D = density of isoflurane (1.5428 $g.ml^{-1}$ at 4°C);

MW ⁄ D = volume for one mole of liquid isoflurane;

Blood ⁄ gas partition coefficient (isoflurane) = 0.97 ±0.01 (0.95–0.98);

According to the ideal gas law, one mole of isoflurane (MW = 184.5 gram) is equal to 22.4 L of isoflurane vapor at one atm. Therefore, 1 ml isoflurane vapor is equal to 8.23 mg at 0°C. As temperature increases from 0 to 37°C, 1 ml isoflurane vapor should be equal to 7.24 mg, according to the ideal gas law: The mass of 1 ml isoflurane at 37°C = (273°K/273+37°K) × 8.23 mg. The piglet's blood/gas partition coefficient was obtained using the method described in the paragraph above. For example, if $\lambda_{b/g}$ is assumed to be 0.97, an equilibrated sample of 100 ml isoflurane gas and 100 ml of blood (each at 1% concentration) would contain 7.24 mg and 7.02 mg of isoflurane, respectively at 37°C (one atm). Therefore, it can be calculated that 1 ml of 1% isoflurane in blood would contain isoflurane 0.0702 mg, which was injected into a 10 ml vial containing 1 ml of blank blood. After equilibration, the measured GC counts represent 1% isoflurane in blood.

V. Calibration curve for measuring blood isoflurane concentrations

A bottle of isoflurane was immersed in a water bath at 4°C for 1 hour before use. Five known amounts of isoflurane liquid were drawn up using a microsyringe (Hamilton 0.5 µl syringe; No. 86259) and injected into five 10-ml glass vials. Each vial contained 1 ml of the piglet's blank blood at 4°C, and was allowed to equilibrate for 30 minutes. A linear relationship between peak isoflurane

counts (y-axis) and isoflurane concentrations (x-axis) was established with the correlation coefficients ranging between 0.9995 and 0.9998, was obtained. The analytical range for the isoflurane concentration was 0.32% to 7.95%. The concentration of isoflurane in blood was then determined from this calibration curve. Similar procedures were followed for the determination of isoflurane concentration in CSF. The coefficients of variation for the intraday accuracy and isoflurane detection precision were 3.4% and 6.0%, respectively.

Reference

Smith MA, Sapsed-Byrne SM, Lockwood GG. A new method for measurement of anaesthetic partial pressure in blood. Br J Anaesth 1997 Apr;78(4):449–452.
