# Supplementary material for: Pharmacokinetics of isoflurane uptake and disposition in piglets
Source: Front Pharmacol. 2026 Oct 15;17:1858313. doi: 10.3389/fphar.2026.1858313 (PMC13716762; doi:10.3389/fphar.2026.1858313)
Supplement: Supplementary file 2 [file DataSheet2.pdf]

Supplementary Materials

S2 Compartment models

One-compartment IV-Infusion, no lag time, 1st order elimination

Model Scheme:

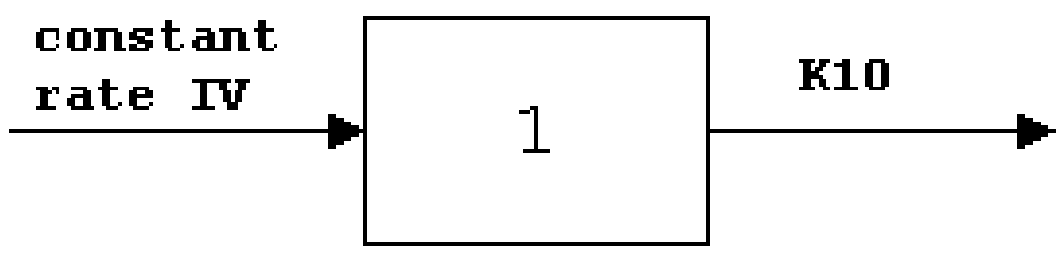

Model Equation:

Y(T) = (D/TI)/V/K10*(EXP(-K10*TSTAR)

-EXP(-K10*T))

Where, *Y* represents the end-tidal, arterial blood, and pulmonary artery blood concentrations; TI = infusion length, *TSTAR = T – TI for T>TI; TSTAR = 0 for T≤TI* ;

Two-compartment IV-Infusion, no lag time, 1st order elimination

Model Scheme:

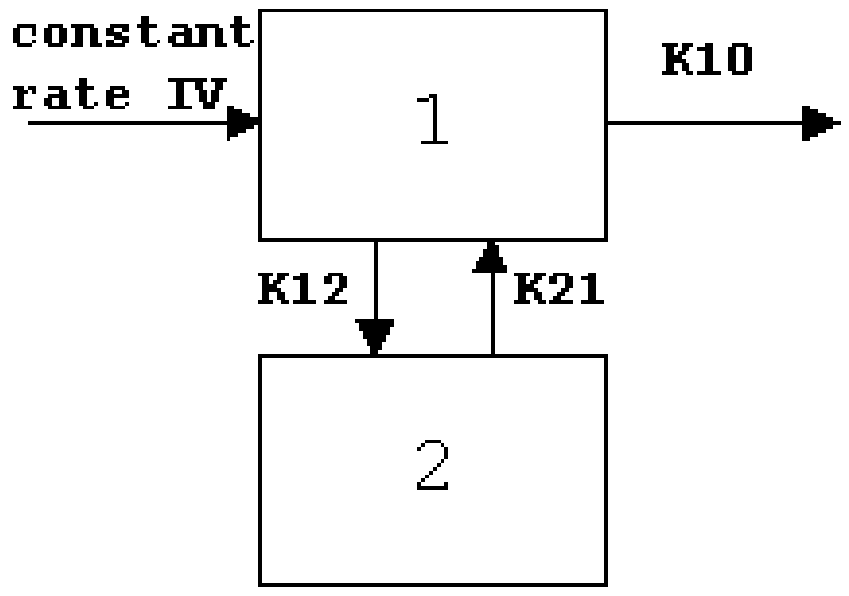

Model Equation:

Y(T)=A_1*(EXP(-ALPHA*T)-EXP(-ALPHA*TSTAR))

+B_1*(EXP(-BETA*T)-EXP(-BETA*TSTAR))

Where, *Y* represents the end-tidal, arterial blood, and pulmonary artery blood concentrations; TI = infusion length, *TSTAR = T – TI for T>TI; TSTAR = 0 for T≤TI* ;

$$A_1 = \frac{D}{TI(V)} \times \frac{(K_{21} - Alpha)}{(Alpha - Beta)Alpha}$$

$$B_1 = \frac{-D}{TI(V)} \times \frac{(K_{21} - Beta)}{(Alpha - Beta)Beta}$$

Three-compartment IV-Infusion, no lag time, 1st order elimination

Model Scheme:

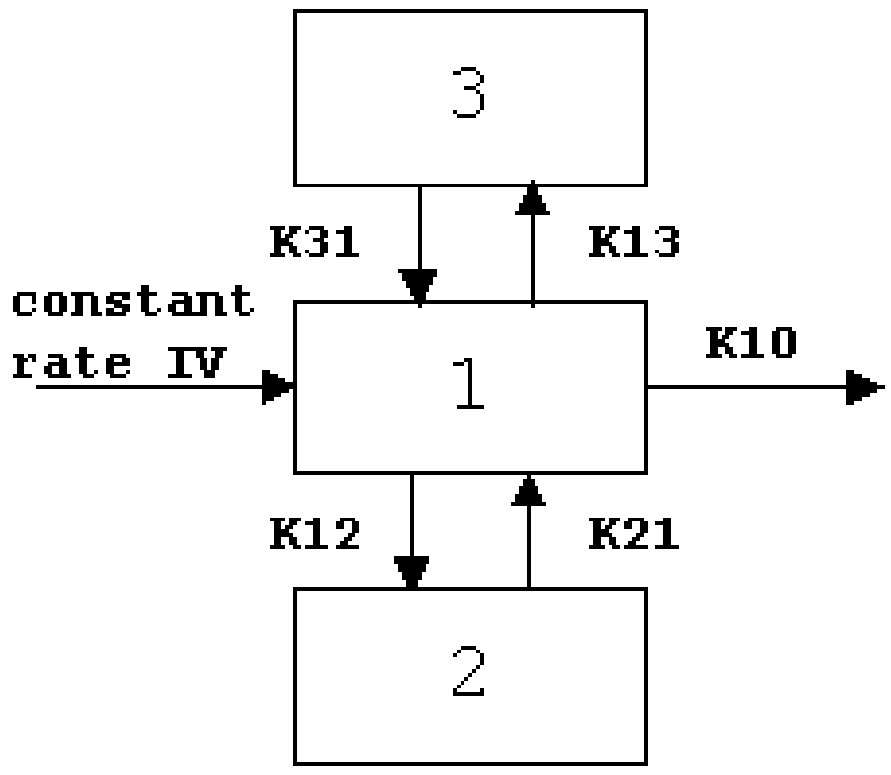

Model Equation:

Y(T)=A_1*(EXP(-ALPHA*T)-EXP(-ALPHA*TSTAR))

+B_2*(EXP(-BETA*T)-EXP(-BETA*TSTAR))

+C_3*(EXP(-GAMMA*T)-EXP(-GAMMA*TSTAR))

Where, *Y* represents the end-tidal, arterial blood, and pulmonary artery blood concentrations; TI = infusion length, *TSTAR = T – TI for T>TI; TSTAR = 0 for T≤TI* ;

$$A_1 = \frac{(\frac{D}{TI})(K_{21} - Alpha)(K_{31} - Alpha)}{V(Gamma - Alpha)(Beta - Alpha)Alpha}$$

$$B_1 = \frac{(\frac{D}{TI})(K_{21} - Beta)(K_{31} - Beta)}{V(Gamma - Beta)(Alpha - Beta)Beta}$$

$$C_1 = \frac{(\frac{D}{TI})(K_{21} - Gamma)(K_{31} - Gamma)}{V(Alpha - Gamma)(Beta - Gamma)Gamma}$$
