# Supplementary material for: Pharmacokinetics of isoflurane uptake and disposition in piglets
Source: Front Pharmacol. 2026 Oct 15;17:1858313. doi: 10.3389/fphar.2026.1858313 (PMC13716762; doi:10.3389/fphar.2026.1858313)

frontiers

Supplementary Materials

S3 Model selection criteria by Akaike Information Criterion (AIC)

TABLE S1. Isoflurane arterial blood concentration (Aiso)

ITEM	Pig	1-compartment	2-Compartment	3-Compartment
**AIC**	1	-11.83	-59.94	-71.1
	2	0.93	-56.54	-83.88
	3	-2.6	-18	-23.6
	4	-9.74	-60.08	-70.86
	5	-15.68	-30.1	-37.44
	6	0.64	-35.3	-41.44
	7	-4.13	-62.19	-77.67
	**Mean**	**-6.06**	**-46.02**	**-58.00**
	**SD**	**6.44**	**17.87**	**23.36**
	**Compare**	***P*-value**		
	1 vs. 2	< 0.001		
	1 vs. 3	< 0.001		
	2 vs. 3	0.214		

TABLE S2. Isoflurane pulmonary artery blood concentration (PAiso)

ITEM	Pig	1-compartment	2-Compartment	3-Compartment
**AIC**	1	-15.09	-63.65	-69.81
	2	-7.7	-50.37	-53.87
	3	-19.3	-31.16	-34.14
	4	-13.48	-76.68	-78.7
	5	-20.52	-39.06	-48.89
	6	-6.99	-60.88	-62.64
	7	-9.71	-43.21	-57.97
	**Mean**	**-13.26**	**-52.14**	**-58.00**
	**SD**	**5.41**	**15.84**	**14.48**
	**Compare**	**P-value**		
	1 vs. 2	< 0.001		
	1 vs. 3	< 0.001		
	2 vs. 3	0.402		

TABLE S3. Isoflurane end-tidal concentration (ETiso)

ITEM	Pig	1-compartment	2-Compartment	3-Compartment
**AIC**	1	-8.87	-37.19	-34.99
	2	-9.27	-30.15	-26.09
	3	1.03	-6.32	-3.91
	4	-5.46	-53.07	-52.12
	5	-11.67	-29.46	-34.19
	6	-10.4	-33.84	-40.11
	7	-7.09	-58.29	-66.49
	**Mean**	**-7.39**	**-35.47**	**-36.84**
	**SD**	**4.24**	**17.08**	**19.72**
	**Compare**	**P-value**		
	1 vs. 2	0.011		
	1 vs. 3	0.007		
	2 vs. 3	0.986		

Figure S1. The fitting model of isoflurane arterial blood concentrations (Aiso)

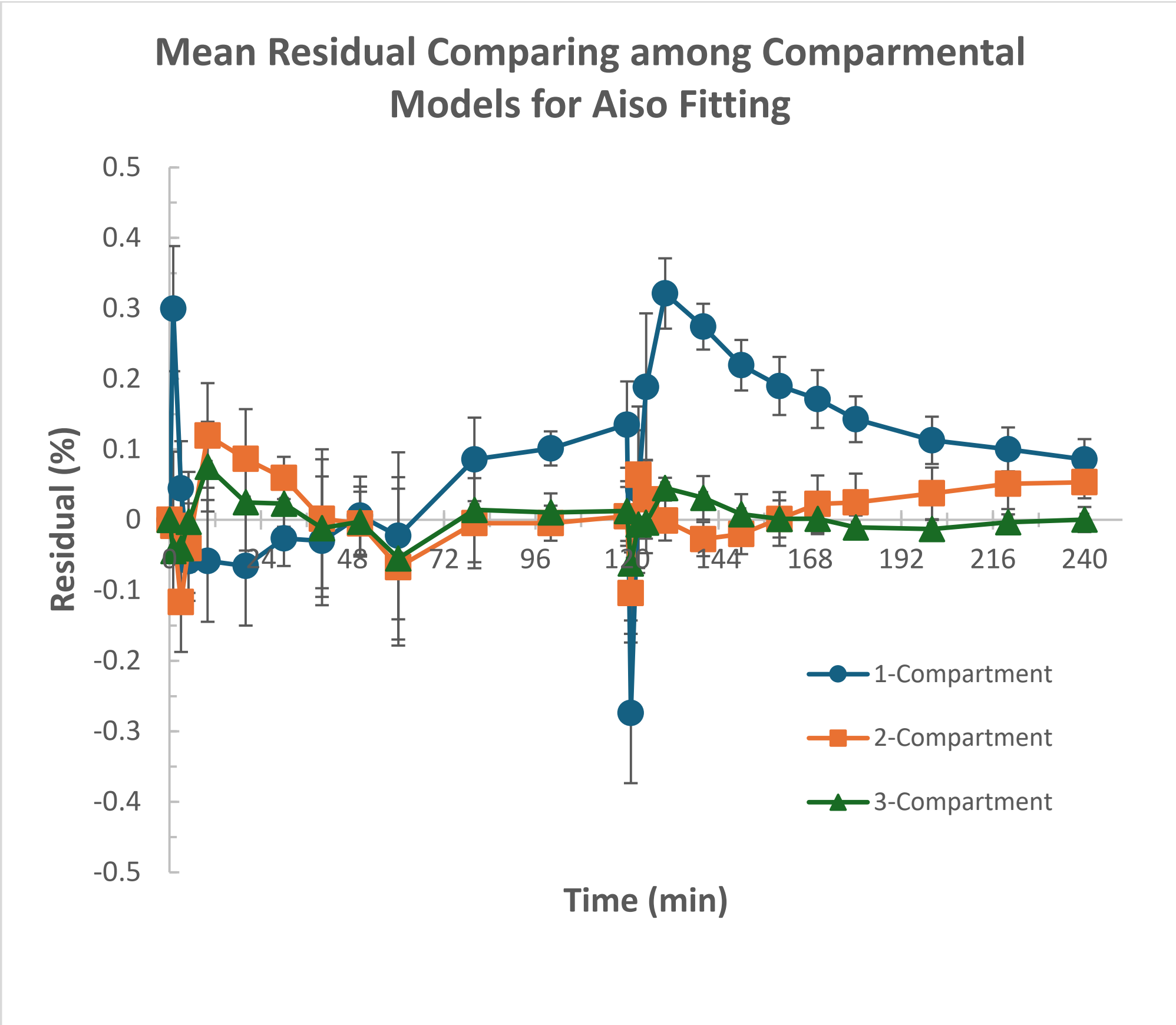

Figure S2. The fitting model of isoflurane pulmonary artery blood concentrations (PAiso)

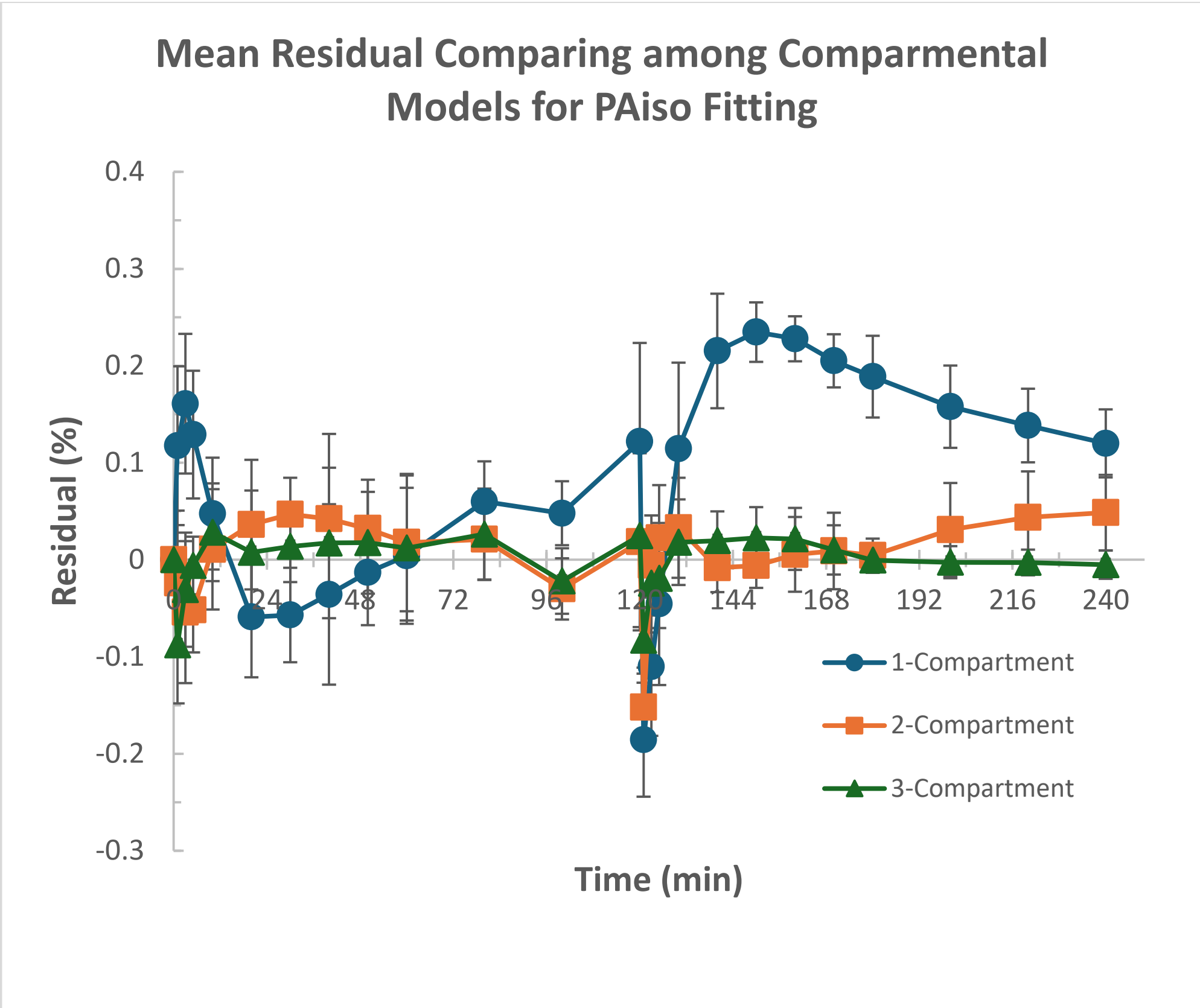

Figure S3. The fitting model of isoflurane end-tidal concentrations (ETiso)

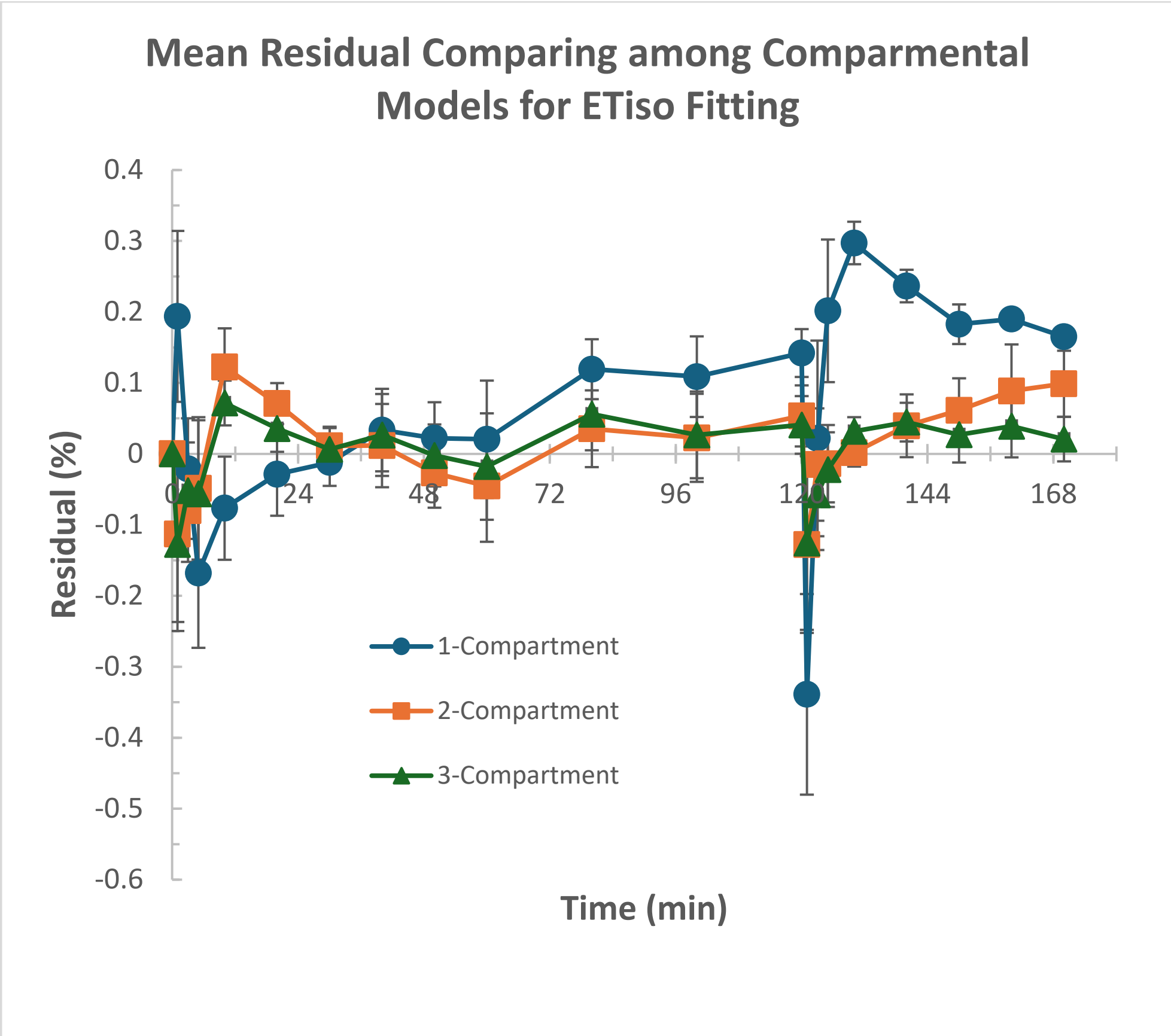

Supplement: Supplementary file 3 [file DataSheet3.pdf]
