# Supplementary material for: Pharmacokinetics of isoflurane uptake and disposition in piglets
Source: Front Pharmacol. 2026 Oct 15;17:1858313. doi: 10.3389/fphar.2026.1858313 (PMC13716762; doi:10.3389/fphar.2026.1858313)

Supplementary Materials

Table S4. The fitting parameters of the end-tidal, arterial blood, and pulmonary artery blood concentrations during the uptake and disposition phases.

Parameters	End-tidal concentration	Arterial blood concentration	Pulmonary artery blood concentration
A_1 (%)	-1.086 (0.150)[a]	-1.020 (0.129)[b]	-0.754 (0.208)
B_1 (%)	-0.603 (0.124)	-0.547 (0.940)	-0.581 (0.133)
Alpha ($1.min^{-1}$)	1.37 (0.27)[a]	1.15 (0.30)[b]	0.48 (0.16)
Beta ($1.min^{-1}$)	0.056 (0.02)[b]	0.03 (0.01)	0.02 (0.01)
Alpha half-life, min	0.52 (0.10)[a]	0.64 (0.18)[a]	1.60 (0.50)
Beta half-life, min	14.43 (6.43)[a]	30.29 (10.62)[b]	45.88 (15.31)
Mean residence time, min	7.50 (2.23)[a]	15.54 (4.35)[a]	28.87 (7.70)
K_{21} ($1.min^{-1}$)	0.0884 (0.0422)[b]	0.0394 (0.0154)	0.0346 (0.0284)
V (%/%)	0.0057 (0.0012)[a]	0.0075 (0.0019)[a]	0.0250 (0.0051)
Maximal concentration, %	1.68 (0.08)[b]	1.53 (0.06)[b]	1.24 (0.08)
Area under the curve, % × min	202.7 (8.77)[b]	188.01 (8.69)[c]	160.19 (14.26)

Values are presented as means (± standard deviations).

Biexponential equation: *Y(T) = A_1*(EXP(-ALPHA*T)-EXP(-ALPHA*TSTAR)) +B_1*(EXP(-BETA*T)-EXP(-BETA*TSTAR))*, y represents the end-tidal, arterial blood, and pulmonary artery blood concentrations; TI = infusion length, *TSTAR = T – TI for T > TI (<u>Disposition phase</u>); TSTAR = 0 for T ≤ TI (<u>Uptake phase</u>)*;

$$A_1 = \frac{D}{TI\ (V)} \times \frac{(K_{21} - Alpha)}{(Alpha - Beta)\ Alpha}$$

$$B_1 = \frac{-D}{TI\ (V)} \times \frac{(K_{21} - Beta)}{(Alpha - Beta)\ Beta}$$

a, $P < 0.001$; b, $P < 0.01$; c, $P < 0.05$, compared with the significance levels of the pulmonary artery blood concentrations.

Figure S4. Comparison of observed (open circles) and model-predicted (dotted lines) isoflurane concentration (%)–time curves.

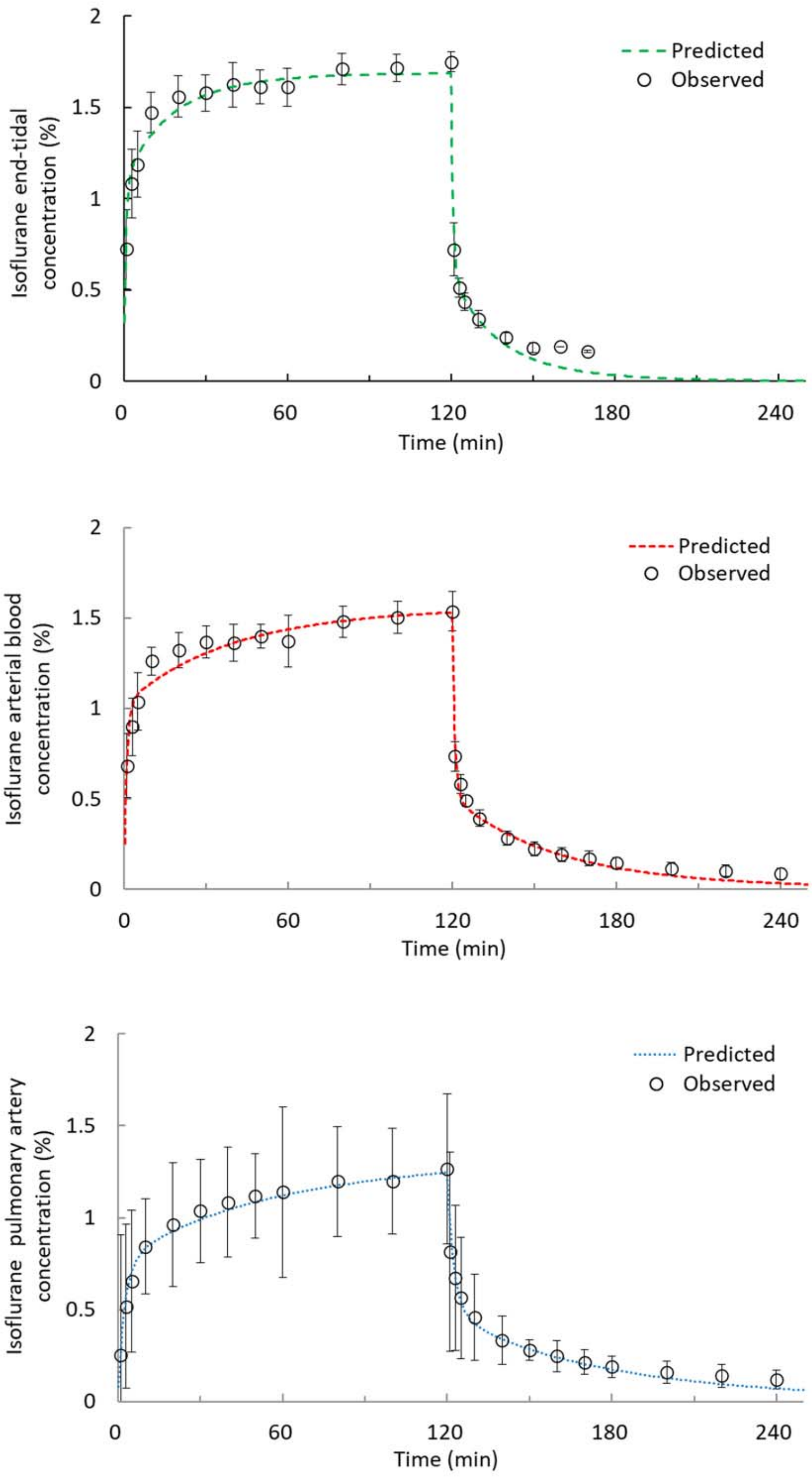

Supplement: Supplementary file 4 [file DataSheet4.pdf]
